# Simulated heatwave and fishing stressors alter corticosteroid and energy balance in neonate blacktip reef sharks, *Carcharhinus melanopterus*.

**DOI:** 10.1093/conphys/coab067

**Published:** 2021-08-26

**Authors:** Alexandra N Schoen, Ian A Bouyoucos, W Gary Anderson, Catharine J Wheaton, Serge Planes, Natalie D Mylniczenko, Jodie L Rummer

**Affiliations:** 1 Department of Biological Sciences, University of Manitoba, 50 Sifton Road, Winnipeg, Manitoba, R3T 2N2, Canada; 2 Australian Research Council Centre of Excellence for Coral Reef Studies, James Cook University, Townsville, Queensland, 4811, Australia; 3 PSL Research University, EPHE-UPVD-CNRS, USR 3278 CRIOBE, Université de Perpignan, 58 Avenue Paul Alduy, 66860 Perpignan Cedex, France; 4 Disney Animals, Science and Environment, Disney’s Animal Kingdom® and the Seas with Nemo and Friends®, Lake Buena Vista, FL 32830, USA; 5 Laboratoire d’Excellence ‘CORAIL’, EPHE, PSL Research University, UPVD, CNRS, USR 3278 CRIOBE, Papetoai, Moorea, French Polynesia; 6 College of Science and Engineering, James Cook University, Townsville, Queensland, 4811, Australia

**Keywords:** stress, metabolite, glucose, elasmobranch, corticosteroid

## Abstract

The increasing frequency and duration of marine heatwaves attributed to climate change threatens coastal elasmobranchs and may exacerbate existing anthropogenic stressors. While the elasmobranch stress response has been well studied, the role of the unique corticosteroid—1α-hydroxycorticosterone (1α-OHB)—in energy balance is not understood. Therefore, 1α-OHB’s utility as a stress biomarker in elasmobranch conservation physiology is equivocal. Here, we analyse the roles of corticosteroids, 1α-OHB and corticosterone, and metabolites, glucose and 3-hydroxybutyrate (3-HB), in response to stress in a protected tropical shark species, the blacktip reef shark (*Carcharhinus melanopterus*). Wild-caught neonates were exposed to ambient (27°C) or heatwave conditions (29°C) and subsequently a simulated fishing stressor (1 min air exposure). Blood samples were taken prior to temperature exposure, prior to air exposure, and 30 min, 1 h, 24 h, and 48 h post-air exposure at treatment temperatures. Plasma 1α-OHB was elevated for 48 h in 27°C-exposed sharks but declined over time in 29°C-exposed sharks. Plasma 1α-OHB was not correlated with either metabolite. Plasma glucose was higher and plasma 3-HB was lower in 29°C-exposed sharks. In a separate experiment, blood samples were collected from both neonate and adult sharks immediately following capture and again 5 min later, and analysed for corticosteroids and metabolites. Plasma 1α-OHB increased in neonates within 5 min, but neonates displayed lower plasma 1α-OHB and higher glucose concentrations than adults. We conclude that 1α-OHB does not serve as a classic glucocorticoid role in *C. melanopterus* under these stressors. Furthermore, we show for the first time, ontogenetic differences in plasma 1α-OHB. Ultimately, our findings provide insights into hormonal control of energy mobilization during stress in *C. melanopterus*, particularly during simulated heatwave conditions, which seem to alter both endocrine and energy mobilization. Further work is needed to determine the utility of 1α-OHB as a biomarker for the mobilization of energy during a stress event in elasmobranchs.

## Teaser

Lay Summary

In tropical nearshore habitats, thermal stressors may exacerbate existing stressors in sharks. Here, we investigated the mobilization of plasma corticosteroids and metabolites in response to a heatwave stress and air exposure in the blacktip reef shark, *Carcharhinus melanopterus.* Thermal stress alters corticosteroid and metabolite mobilization; although, these measurements are not correlated.

## Introduction

Global mean surface ocean temperatures are expected to increase on average by 3.7°C (range, 2.6–4.8°C) by 2100 under a ‘business as usual’ model (IPCC, 2019), with the greatest degree of surface warming occurring in subtropical and tropical regions (Collins *et al*., 2013). In addition, tropical regions are already experiencing an increase in the frequency and duration of marine heat waves, phenomena that rapidly raise sea surface temperatures by up to 3°C for several days to weeks (Frölicher *et al.,* 2018; Frölicher and Laufkötter, 2018). This degree of warming is particularly problematic when considering that ocean temperatures in the tropics can vary seasonally by only 4–6°C (Rummer and Munday, 2017). Consequently, tropical fishes may be more strongly impacted by marine heatwaves than temperate species (Vinagre *et al.,* 2016; Vinagre *et al.,* 2018), given their relatively restricted thermal tolerance and narrow temperature range for metabolic performance (Tewksbury *et al.,* 2008). The increasing frequency of marine heatwaves therefore presents an immediate challenge to tropical fishes; individuals must adapt to or avoid unfavourable thermal conditions (Habary *et al.,* 2017; Payne *et al.,* 2018). Failure to do so may result in significant sublethal impacts to health if not mortality (Bernal *et al.,* 2020; Johansen *et al.*, 2021).

A number of tropical species already appear to be living close to or at their thermal maxima (Rummer *et al.,* 2014), particularly early life history stages that make use of shallow and dynamic nearshore environments where daily temperatures are often highly variable and can rise to 35–36°C (Knip *et al.,* 2010; Bouyoucos *et al.,* 2020). In the tropics, neonates and juveniles of certain shark species can exploit nearshore habitats for increased access to food, shelter from predators and possibly optimal abiotic conditions (i.e. salinity, dissolved oxygen, etc.) (Heupel *et al.,* 2007; Knip *et al.,* 2010). Thus, the potential for neonate and juvenile sharks to experience significant thermal stress in nearshore habitats coupled with life history strategies, such as low fecundity rates, and long generation times (Frisk *et al.,* 2001), make many sharks exceptionally vulnerable to population decline (Frisk *et al.,* 2001; Chin *et al.,* 2010). Specifically, increasing ocean temperatures and fishing pressures act as two of the most significant stressors tropical elasmobranchs face, especially when acting synergistically (Chin *et al.,* 2010; Dulvy *et al.,* 2014). Studies suggest that high temperatures exacerbate physiological stress associated with recreational catch-and-release fishing or artisanal gill-net fishing in juveniles of several tropical shark species (Danylchuk *et al.,* 2014; Bouyoucos *et al.,* 2018a). This is particularly problematic in shallow, coastal shark habitats, where fishing pressure is thought to be high owing to accessibility of these ecosystems by humans (Knip *et al.,* 2010). Together, this suggests that anthropogenic stressors, when coupled with thermal stress, may potentially compromise homeostatic function via persistent activation of the stress axis and therefore impact overall biological fitness.

The vertebrate endocrine stress axis involves a cascade of events typically resulting in increased circulating corticosteroids and often (but not always; see Romero, 2004; Romero *et al.*, 2009; Romero and Beattie, 2021, for a review) an immediate but transient liberation of glucose from internal stores as a primary fuel source to promote restoration of homeostasis during and after a stressful event. Glucocorticoids classically regulate energy balance in vertebrates predominantly through mobilizing glucose (Krebs *et al.,* 1963; Patent 1970; Newsholme and Start, 1973; Pardridge, 1983; Wendelaar Bonga, 1997). While cortisol is the dominant glucocorticoid in teleosts (Wendelaar Bonga, 1997; Barton, 2002; Gorissen and Flik, 2016), 1α-hydroxycorticosterone (1α-OHB) (Idler and Truscott, 1966) is the presumptive glucocorticoid in elasmobranchs (Hazon and Henderson, 1985; Anderson, 2012). However, a glucocorticoid role for 1α-OHB in elasmobranchs remains equivocal. In a recent paper, the Pacific spiny dogfish (*Squalus acanthias suckleyi*) did not exhibit a correlation between 1α-OHB and glucose, and only a weak relationship between 1α-OHB and the ketone body, β-hydroxybutyrate (β-HB; biologically equivalent to 3-HB). This suggests that 1α-OHB may still be involved in mobilization of energy in the spiny dogfish through ketones as an alternative fuel source to glucose (Schoen *et al.,* 2021). Interestingly, there was a positive correlation, albeit weak, between circulating levels of corticosterone and glucose (Schoen *et al.*, 2021), which is consistent with previous studies in elasmobranchs that show a strong increase in glucose following a stressful event (Patent, 1970; Manire *et al.,* 2007; Brinn *et al.,* 2012); indeed, corticosterone may act as a classic glucocorticoid in some elasmobranchs.

There is interest in measuring 1α-OHB in elasmobranchs and evaluating its utility as a stress biomarker (Skomal and Mandelman, 2012; Madliger *et al*.*,* 2018). Unfortunately, the lack of a consistent and reproducible 1α-OHB assay prior to 2018 (Wheaton *et al.,* 2018) has limited the ability to directly measure and assess the role of 1α-OHB in the stress response of elasmobranchs. Consequently, the majority of studies examining the physiological consequences of thermal stress in elasmobranchs report changes in blood metabolite and ion balance during the secondary stress response, rather than measurement of the endocrine response. Thermal stress has been shown to impact metabolite mobilization in elasmobranchs, where circulating glucose usually increases (Cicia *et al.,* 2012; Guida *et al.,* 2016; Bouyoucos *et al.,* 2018a; Bouyoucos *et al.,* 2018b) and circulating β-HB has been shown to decrease (French *et al.,* 2015) with increasing temperatures. In addition to glucose as a metabolic fuel, elasmobranchs rely heavily on β-HB as an extrahepatic fuel source (Zammit and Newsholme, 1979; Richards *et al.,* 2003; Speers-Roesch and Treberg, 2010). However, few studies have investigated ketone mobilization in response to thermal stress in elasmobranchs (French *et al.,* 2015). While changes in metabolite concentrations, and even corticosteroid concentrations (i.e. corticosterone), in sharks following a stressor are assumed to be positively correlated with changes in circulating 1α-OHB in the acute phase of the stress response, this notion remains to be validated.

A further step towards evaluating 1α-OHB as a stress biomarker includes testing for ontogenetic effects. Indeed, studies investigating thermal stress in elasmobranchs have traditionally focused on a particular life stage. However, it is well recognized in teleosts that the endocrine stress response may not be fully developed until several weeks post-hatch (Wendelaar Bonga, 1997) and is likely dependent on associated tissue development (Barry *et al.,* 1995; Jentoft *et al.,* 2002). To date, investigations on the ontogeny of hormone development in elasmobranchs have largely focused on reproductive hormones (Rasmussen and Gruber, 1993; Manire *et al.,* 1999; Sulikowski *et al.,* 2005; Sulikowski *et al.,* 2006; Awruch, 2013), with limited examination of thyroid hormones (Crow *et al.,* 1999) and α-melanocyte-stimulating hormone (Chiba and Oka, 2002). In regards to development of the stress axis and potential glucocorticoids, corticosterone was shown to increase in male lemon sharks (*Negaprion brevirostris*) with increasing precaudal length (a proxy for age), but not in juvenile whitetip reef sharks (*Triaenodon obesus*) over 36 months in captivity (Rasmussen and Crow, 1993). To our knowledge, no studies to date have examined the effect of age on circulating levels of 1α-OHB. Similarly, few studies have examined the effect of age on metabolites in elasmobranchs following exposure to a stressor. Fork length, which can be used as a coarse (but limited) estimate of age and/or developmental stage, was not determined to be an effective predictor of plasma secondary stress parameters in several carcharhinid species (Mandelman and Skomal, 2009), and total length was not found to be related to glucose concentrations in juvenile blacktip sharks (*Carcharhinus limbatus*) following capture (Manire *et al.,* 2001). By comparison, plasma glucose concentrations were slightly higher in young-of-the-year smalltooth sawfish (*Pristis pectinate*) when compared to adults following capture stress, but the difference was not significant and did not account for capture method (Prohaska *et al.,* 2018).

Here, we tested the hypothesis that circulating levels of 1α-OHB would change in response to a simulated stressor and serve a role in energy mobilization in an elasmobranch species of conservation concern, the blacktip reef sharks (*C. melanopterus*) (MacNeil *et al.,* 2020). Thermal stress was selected as the stressor to provide insight into the physiological changes that neonatal *C. melanopterus* may experience during a heatwave event and while utilizing shallow, nearshore habitats. We chose an additional stressor—air exposure—to simulate a fishing stress event (e.g. bycatch) that these sharks may be challenged with while already under thermal stress. We predicted that circulating levels of the corticosteroids, 1α-OHB and corticosterone, and the metabolites, glucose and 3-HB, would be altered by the simulated stressors and positively correlated with temperature. Additionally, we tested the hypothesis that there would be a relationship between age and the corticosteroid and metabolite response in blacktip reef sharks following an acute, simulated fishing stressor. We predicted that the neonates would have a muted response in comparison to adults, as the relevant endocrine machinery may not yet be fully developed in neonates (Wendelaar Bonga, 1997). The data presented herein provides the first insight into how a representative elasmobranch may regulate energy balance during compounding thermal and anthropogenic stress events. Further, our study represents a first step towards evaluating 1α-OHB as a stress biomarker for sharks while considering temperature and ontogeny. Given that glucocorticoids are informative and available stress biomarkers in the majority of vertebrate taxa (Madliger *et al.,* 2018), there is a need to develop this tool for elasmobranch conservation physiology assessments.

## Materials and Methods

### Ethical approval

All protocols described were approved by the James Cook University Animal Ethics Committee Protocol A 2394. Sharks were collected under the Arrêté 11 491 of the French Polynesian Ministère de la Promotion des Langues, de la Culture, de la Communication, et de l’Environnement.

### Laboratory shark collection and care

Neonate *C. melanopterus* (visible umbilical scar, <4 weeks of age; Chin *et al.,* 2015; total length, 522–618 mm; *n* = 12; 7 male and 5 female) were collected within 50 m from the shoreline around the island of Moorea, French Polynesia, during November 2019. Sharks were caught in monofilament gillnets (50 m long × 1.5 m deep with 5 cm mesh) between 17:00–22:00 and immediately removed from nets within 5 min. Morphometric data were collected, and each individual was internally tagged with passive integrated transponder tags ~1 cm adjacent to the dorsal fin (Biolog-id SAS; Paris, France) to prevent repeat sampling. Sharks were isolated in flow-through mesh bags to allow for the capture of additional sharks, but for no longer than 1 h post-capture. Sharks were then transferred to the Centre de Recherches Insulaires et Observatoire de l’Environnement (CRIOBE) holding facility by vehicle in 200-l insulated coolers with aerated seawater (up to three sharks per cooler); transport took no longer than 30 min.

A maximum of six sharks were maintained at any given time across two 1250-l circular flow-through tanks (three sharks per tank) fitted with continuous aeration and filtered flow-through seawater supplied from a pump collecting water offshore. Sharks were given at least 1 week in the holding tanks to recover from capture prior to experimentation during which time they were fed fresh tuna (*Thunnus spp.*) *ad libitum* every second day. Following experimentation (a total of 120 h), sharks were released back to their original capture site.

### Thermal exposure

**Figure 1:**
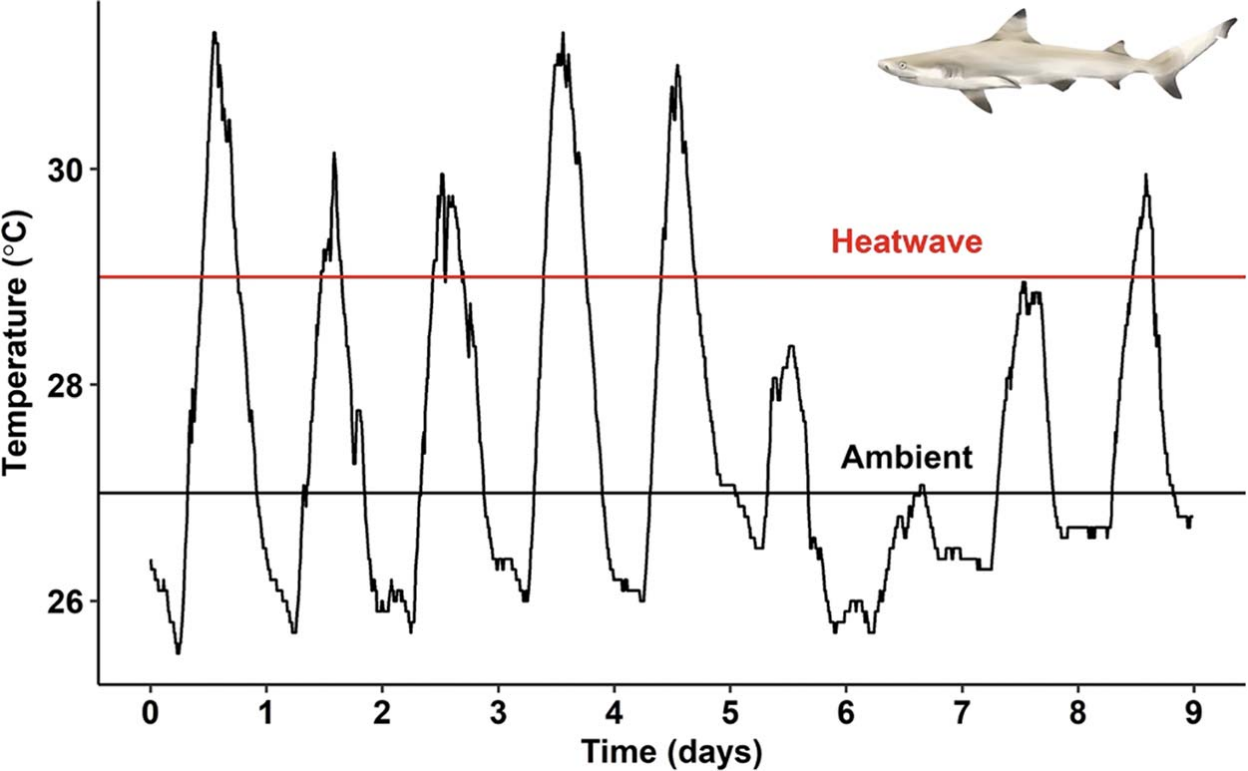
Environmental temperature recorded with a HOBO data-logger off the coast of Papetō’ai, Moorea, between 7 November and 16 November 2019. Experimental ambient (27°C; black line) and heatwave (29°C; red line) are labelled.

Environmental temperatures were recorded using temperature data-loggers (HOBO UA-002-64, Onset Computer Corporation, Bourne, MA, USA) deployed at 10 sites around Moorea where neonatal *C. melanopterus* are commonly collected/observed. One or two loggers were deployed at each site no more than 50 m from shore. A nearly continuous temperature record for those ten sites exists since November 2015, and this record consists of temperature measurements every 10 min. During this study period, environmental temperature ranged between 25.5°C and 31.3°C (Fig. 1).

Sharks were assigned to one of two temperature groups: 27°C or 29°C (*n* = 6 per treatment) with 27°C representing ambient environmental temperatures at the time of experimentation (Fig. 1; black line) and 29°C representing a short-term heatwave scenario (Fig. 1; red line). After 7 days of habituation to the holding tanks at 27± 0.5°C, and prior to increasing water temperatures (for the 29°C treatment group), a blood sample was taken by caudal vessel puncture from all sharks. To do this, an individual shark was removed from its respective holding tank, held ventral side up with the gills and mouth submerged to induce tonic immobility, and a blood sample (1 ml) was taken from the caudal vessel using a 23-gauge needle connected to a heparinized (100 IU ml^−1^ ammonium heparin) 3-ml syringe. This procedure took less than 1 min for each animal and served as the baseline sample for subsequent analyses. Sharks assigned to the 27°C temperature group were maintained for another four days, while the temperature of the tanks for sharks assigned to the 29°C group was increased by 0.5°C per day over 4 days (i.e. + 2°C, to reach 29°C), which is a previously established temperature protocol in this population (Bouyoucos *et al.,* 2020). After 4 days, food was withheld for the remaining duration of experimentation and all sharks were maintained at their target temperatures (e.g. 27 or 29°C) for the following 48 h. At this point, another 1-ml blood sample was taken as a time-0 sample. Target temperatures were maintained for the duration of experimentation over the following 2 days.

### Air exposure

Immediately following the time-0 sample, sharks were individually collected with handheld nets and held out of water for 1 min to simulate the air exposure that can occur during a fisheries interaction. Sharks were then released back into their respective holding tanks. Sharks were repeatedly recaptured by hand so that subsequent 1 ml blood samples were taken at 30 min, 1, 24 and 48 h post-air exposure. In total, each shark was sampled six times over 96 h.

### Wild-caught sharks

Wild neonates (visible umbilical scar, <4 weeks of age; Chin *et al.,* 2015; total length, 526–600 mm; *n* = 6; 4 male and 2 female) were caught via either hook and line (*n* = 2) or gillnet (*n* = 4). Wild adults (1020–1512 mm; *n* = 6; 3 male and 3 female) were hand caught by hook and line using fresh tuna as bait. For both age classes and capture techniques, the duration of capture was less than 2 min. Immediately after capture, a 1 and 3 ml blood sample was taken from the neonates and adults, respectively, in a similar manner as described for the laboratory-based experiment, except an 18-gauge needle was used to sample adults. Morphometric data were then collected for each shark over 5 min, and then a second blood sample of equivalent volume was taken from each individual to assess stress parameters over this timeframe. Sharks were restrained by hand and submerged in water for the duration of experimentation and were immediately released following the second blood sample.

### Blood sample processing

All blood samples were immediately stored on ice until further processing, which occurred after no more than 5 min in the laboratory-based sharks and 1 h in the wild-caught sharks (Schwieterman *et al.,* 2019). Samples were transferred to 2 ml snap-cap conical tubes and centrifuged for 3 min at 13000 g to separate plasma from cellular components. The plasma was pipetted into a clean tube and stored at −80°C for later analysis.

### Glucose and 3-HB assays

Plasma glucose concentrations were measured with an enzymatic hexokinase assay, as outlined in Treberg *et al.* (2007) and adapted for a 96-well plate, as outlined in Schoen *et al.* (2021). Plasma 3-HB concentrations were measured using an enzymatic 3-HBDH assay adapted from Schoen *et al.* (2021), where 0.5 IU.ml^−1^ of 3-HBDH was added to each well (instead of ß-HBDH), except control wells, which had an equal volume of deionized water. Samples were analysed spectrophotometrically at 340 nm for 50 min, or to completion, by measuring the conversion of NAD^+^ to NADH. For all assays, an initial validation was conducted using a pool of 4–6 haphazardly chosen samples. A standard dilution, determined by the validation, was used to dilute all samples. However, if a sample fell below (negative control) or above (top standard) the detection range of an assay, samples were appropriately re-diluted and re-analysed. Glucose and 3-HB concentrations were determined by subtracting the first recorded value (immediately following addition of respective enzymes) from the final recorded value (when the enzyme reaction was complete) and interpolating values from the assay specific standard curve. The time variation in adding enzyme to an assay across the 96-well plate was less than 30 s. All assays were analysed at room temperature. Absorbance was measured with a plate reader (Power wave XS2, Biotek) using Gen5 software (Biotek, Winooski, VT, USA).

### 1α-OHB and corticosterone enzyme immunoassay

Corticosteroid extractions and enzyme immunoassay were performed using the methods in Schoen *et al.* (2021) and Wheaton *et al.* (2018). Standards for 1α-OHB and corticosterone were provided by CJW. Samples were extracted, evaporated and then appropriately concentrated (1α-OHB) or diluted (corticosterone) in assay buffer as to remain in the readable range of the standard curves. Samples with concentrations below the detectable limit of the assays were considered to have a zero concentration. All chemicals (purchased from Sigma Aldrich) and equipment remained the same unless otherwise stated. Samples were randomly assorted on the assay plates and respectively assayed in triplicate (1α-OHB) and duplicate (corticosterone). The cross-reactivity of corticosterone standards on the 1α-OHB assay was 16.1% (Wheaton *et al.,* 2018), and the 1α-OHB standards on the corticosterone assay was 15.1%. Extraction efficiencies, determined by comparing a set of corticosteroid-spiked acetonitrile blanks (spiked with respective standard, 1α-OHB = ~33.8 ng.ml^−1^, corticosterone = ~5.9 ng.ml^−1^) that underwent the extraction process with neat standard (same concentrations), of 1α-OHB and corticosterone were 60.9% and 54.6%, respectively. Intra- and inter-assay variations for 1α-OHB were 6.5% and 9.9% (*n* = 8 assays), respectively, and corticosterone intra- and inter-assay variations were 8.8% and 2.8% (*n* = 6 assays), respectively.

### Statistical analyses

All data (glucose, 3-HB, 1α-OHB, and corticosterone) were subject to Grubb’s outlier tests (α = 0.05). Shapiro–Wilks and Levine’s tests were used to assess normality and equal variance, respectively for each of the measured variables. If these assumptions were not met, data were transformed using a rank transformation.

Unpaired Student’s *t*-tests were used to determine the significance of treatments within individual time points in the laboratory-based sharks, between time-0 in the 27°C laboratory-based and wild neonates, between timepoints in the wild-caught sharks and between age groups in the wild-caught sharks. Time-0 in the 27°C laboratory-based and wild neonates were compared to determine if time-0 in the laboratory-based sharks could be considered a true baseline (as a control for laboratory confinement), since both groups of sharks were exposed to ambient temperatures. Linear mixed effects models (‘lmer’ function in ‘lme4’ package) (Bates *et al.,* 2015) with Tukey’s HSD post hoc tests were used to determine the significance of each treatment over time, as compared to baseline values. Shark identification was added to the model as a random effect, thus accounting for repeat sampling of each shark and non-independence (Zeger and Karim, 1991).

Corticosterone values were non-parametric in all datasets except within the 27°C treatment. Therefore, corticosterone values were subject to Wilcox tests instead of Student’s t-tests. The corticosterone values within the 27°C treatment were subject to the same linear mixed effects model with Tukey’s HSD post hoc test previously mentioned.

Correlation plots between corticosteroids and metabolites using samples collected from the laboratory-based sharks only were analysed using Pearson product–moment correlations. All statistical analyses were performed in R v3.5.2, and statistical significance was accepted at α = 0.05.

## Results

### Thermal and air exposure

**Figure 2:**
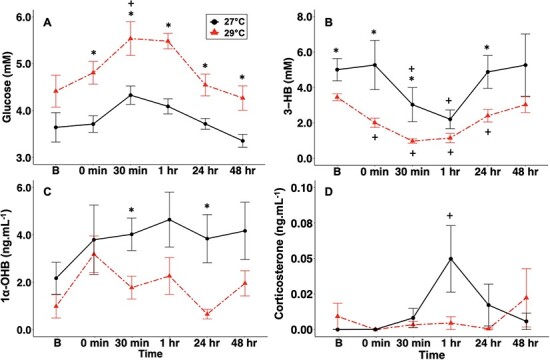
Mean ± SEM (A) glucose (mM), (B) 3-HB (mM), (C) 1α-OHB (ng.ml^−1^) and (D) corticosterone (ng.ml^−1^) concentrations in neonate blacktip reef sharks exposed to 27°C (open triangles, solid line, *n* = 6) or 29°C (filled triangles, dashed line, *n* = 6). Asterisks (*) indicate significance between temperature treatments within a timepoint.

In 29°C-exposed sharks, plasma glucose concentrations significantly increased between baseline and 30 min; this trend was observed in 27°C-exposed sharks as well, but was non-significant. There were no significant increases in plasma glucose concentrations in samples from either the 27°C- or 29°C-exposed sharks following air exposure (Table 1). The 29°C-exposed sharks exhibited significantly higher plasma glucose concentrations when compared to the 27°C-exposed sharks at all timepoints (*t*-test; all *P* < 0.020) except baseline (Fig. 2A).

**Table 1:** *P*-values between baseline (B; pre-temperature treatment) or time-0 (0 min; pre-air exposure) and subsequent timepoints

Measurement	Treatment	B-time-0	B-30 min	B-1 h	B-24 h	B-48 h	Time-0-30 min	Time-0-1 h	Time-0-24 h	Time-0-48 h
Glucose	27°C	1.000	0.069	0.479	1.000	0.863	0.140	0.665	1.000	0.712
	29°C	0.916	0.046^*^	0.067	0.999	0.999	0.417	0.509	0.985	0.735
3-HB	27°C	0.987	0.001	<0.001^*^	0.999	0.747	0.014^*^	<0.001^*^	1.000	0.98
	29°C	<0.001^*^	<0.001^*^	<0.001^*^	0.016^*^	0.787	0.018^*^	0.084	0.828	0.020^*^
1α-OHB	27°C	0.880	0.807	0.545	0.867	0.752	1.000	0.993	1.000	1.000
	29°C	0.054	0.914	0.571	0.998	0.814	0.461	0.847	0.015^*^	0.615
Corticosterone	27°C	0.905	0.185	<0.001^*^	0.120	0.811	0.786	0.027^*^	0.670	1.000
	29°C	1.000	1000	0.088	1.000	0.789	0.371	1.000	1.000	0.371

^*^Significance using linear mixed effect models with Tukey HSD post hocs (Glucose, 3-HB, 1α-OHB, 27°C-exposed corticosterone) or Wilcox tests (29°C-exposed corticosterone).

In the 29°C-exposed sharks, plasma 3-HB concentrations significantly decreased between baseline and time-0 and remained significantly lower than baseline values through 24 h. In the 27°C-exposed sharks, 3-HB significantly decreased at 30 min and remained significantly lower than baseline values at 1 h. Following air exposure, 3-HB significantly increased in both 27°C- and 29°C-exposed sharks. Relative to time-0, 3-HB remained significantly elevated in 27°C-exposed sharks at 1 h and in 29°C-exposed sharks at 48 h (Table 1). The 29°C-exposed sharks exhibited significantly lower plasma 3-HB concentrations than the 27°C -exposed sharks at baseline, time-0, 30 min and 24 h (Fig. 2B; *t*-test; all *P* < 0.040).

**Figure 3:**
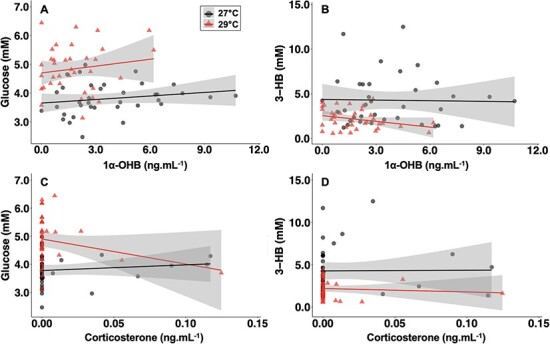
Pearson’s product–moment correlations between metabolite and corticosteroid concentrations in blacktip reef shark neonates exposed to 27°C (triangles) and 29°C (circles) treatments. (A) Correlation between glucose (mM) and 1α-OHB (ng.mL^−1^) concentrations (27°C, *n* = 36, R^2^ = 0.192, *P* = 0.261; 29°C, *n* = 36, R^2^ = 0.159, *P* = 0.374). (B) Correlation between 3-HB (mM) and 1α-OHB (ng.ml^−1^) concentrations (27°C, *n* = 36, R^2^ = −0.029, *P* = 0.899; 29°C, *n* = 36, R^2^ = −0.295, *P* = 0.080). (C) Correlation between glucose (mM) and corticosterone (ng.ml^−1^) concentrations (27°C, *n* = 36, R^2^ = 0.119, *P* = 0.489; 29°C, *n* = 36, R^2^ = −0.252, *P* = 0.139). (D) Correlation between 3-HB (mM) and corticosterone (ng.ml^−1^) concentrations (27°C, *n* = 36, R^2^ = 0.009, *P* = 0.959; 29°C, *n* = 36, R^2^ = −0.082, *P* = 0.635).

In both 27°C- and 29°C-exposed sharks, plasma 1α-OHB increased between baseline and time-0, but this difference was not significant. There was a significant decrease in 1α-OHB in sharks between time-0 and 24 h (Table 1). In the 27°C-exposed sharks, plasma 1α-OHB concentration remained elevated as compared to 29°C-exposed sharks; although, this was only significant at 30 min (*t*-test; *P* = 0.022; DF = 10) and 24 h (Fig. 2C; *t*-test; *P* = 0.037; DF = 10). Plasma corticosterone concentrations were low overall in sharks from both temperature treatments (Fig. 2D), and there was only a significant increase from baseline in corticosterone in the 27°C-exposed sharks at 1 h. There was also a significant increase in corticosterone between time-0 and 1 h in the 27°C-exposed sharks (Table 1). In both 27°C- and 29°C-exposed sharks, there were no correlations between either corticosteroid or metabolite (Fig. 3).

### Wild-caught sharks (neonates versus adults)

**Figure 4:**
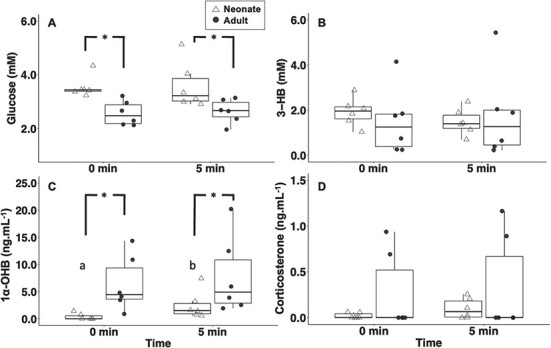
(A) glucose (mM), (B) 3-HB (mM), (C) 1α-OHB (ng.ml^−1^) and (D) corticosterone (ng.ml^−1^) concentrations in wild neonate (open triangles, *n* = 6) and adult (filled circles, *n* = 6) blacktip reef sharks exposed to 5 min. handling stress. Asterisks (*) indicate significance between neonates and adults. Letters indicate significance between timepoints within age groups.

Glucose concentrations were significantly higher in neonates, as compared to adults, at both the time-0 baseline (*t*-test; *P* = 0.003; DF = 10) and 5 min post-stressor (Fig. 4A; *t*-test; *P* = 0.038; DF = 10). In contrast, plasma 1α-OHB concentrations were significantly higher in adults than neonates at both time points (*t*-test; both *P* < 0.050; both DF = 10). In neonates, 1α-OHB concentrations were significantly higher between the time-0 baseline and 5 min post-stressor (Fig. 4C; *t*-test; *P* = 0.006; DF = 10). There were no significant changes in 3-HB or corticosterone between age classes or timepoints (Fig. 4B and D).

### Laboratory versus wild neonates

**Figure 5:**
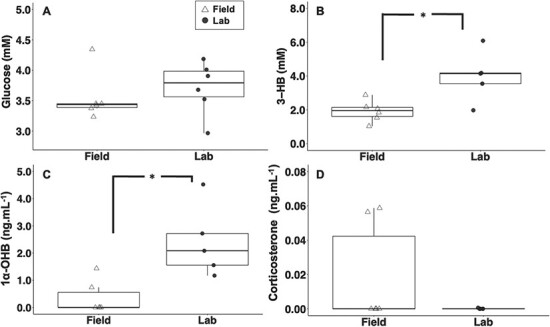
(A) glucose (mM, *n* = 6), (B) 3-HB (mM, field: *n* = 6, laboratory: *n* = 5), (C) 1α-OHB (ng.ml^−1^, field: *n* = 6, laboratory: *n* = 5) and (D) corticosterone (ng.ml^−1^, *n* = 6) concentrations in wild field (open triangles) and laboratory held at 27°C (filled triangles) neonate blacktip reef sharks at time-0 of respective experiments. Asterisks (*) indicate significance between wild and laboratory neonates.

When comparing the 27°C-exposed laboratory-based neonates with the wild-caught neonates, neither plasma corticosterone nor glucose concentrations significantly differed at time-0 (Fig. 5A and D). However, the 27°C-exposed laboratory-based neonates exhibited significantly higher circulating levels of 1α-OHB (*t*-test; *P* < 0.01; DF = 9) and 3-HB (*t*-test; *P* = 0.01; DF = 9) than wild-caught (field) neonates. (Fig. 5B and C).

## Discussion

The overall goal of this study was to investigate the role of the presumptive elasmobranch glucocorticoid, 1α-OHB, as a stress biomarker in neonates of a protected reef shark (*C. melanopterus*). The first objective was to test if 1α-OHB levels changed following exposure to a simulated stressor and determine its role in energy mobilization during the combined temperature (i.e. simulating heatwave conditions) and subsequent air exposure challenges (i.e. simulating a bycatch event). The second objective of this study was to determine differences in the acute phase of the stress response (5 min) between two developmental stages (neonate vs adult). To our knowledge, this is the first study to examine changes in circulating levels of glucose and 3-HB concurrently with the dominant elasmobranch corticosteroids in a tropical shark species, as well as the first time 1α-OHB was measured in neonates and adults of the same species. These findings are a first step in understanding the hormonal and metabolic response of neonate *C. melanopterus* to near-future heatwave conditions, as well as interpreting ontogenetic variations in corticosteroid and metabolite mobilization pathways. This information can help in determining the efficacy of 1α-OHB as a tool for investigating the endocrine stress response and therefore contribute to conservation physiology research in elasmobranchs.

### Thermal and air exposure

The 29°C-exposed (simulated heatwave) sharks exhibited higher plasma glucose and lower 3-HB than the 27°C-exposed (current-day conditions) counterparts. The trend with glucose may not be surprising, however. Increases in circulating glucose with increasing temperature has been documented across a number of elasmobranch species following fishing capture (French *et al.,* 2015; Guida *et al.,* 2016; Bouyoucos *et al.,* 2018a), including in the same population of *C. melanopterus* (Bouyoucos *et al.,* 2018b) as investigated in the present study. Additionally, seasonal increases in glucose were noted in Atlantic sharpnose sharks (*Rhizoprionodon terraenovae*; Hoffmayer *et al.,* 2012), lesser-spotted dogfish (*Scyliorhinus canicula*; Gutiérrez *et al.,* 1987) and little skates (*Leucoraja erinacea*; Cicia *et al.,* 2012), which may indicate that plasma glucose simply rises during warmer months, but this trend could also result from increased foraging during warmer months. Nevertheless, a rise in plasma glucose with warmer temperatures seems to be a consistent trend among elasmobranchs following fishing capture. Furthermore, to our knowledge, this is the second account of a change in a ketone body with temperature in an elasmobranch species, with the first being a decrease in plasma β-HB over a 1.5°C increase in sea surface temperature following capture in shortfin mako sharks (*Isurus oxyrinchus*; French *et al.,* 2015). Valls *et al.* (2016) also recorded a seasonal decrease in 3-HB in *S. canicula* adult males, but the effect of temperature was not directly tested. In *C. melanopterus* in the present study, the 29°C-exposed sharks exhibited significantly lower circulating 3-HB as well as a smaller change between time-0 and 30 min than their 27°C-exposed counterparts, which suggests that an increase in temperature reduced the concentration of plasma 3-HB available for consumption in this species. In European eel (*Anguilla anguilla*) hepatocytes, acetoacetate (the biosynthetic precursor of 3-HB or β-HB) in the cell media decreased with increasing acclimation temperature over 3 weeks (Jankowsky *et al.,* 1984) and β-HB oxidation increased in hepatic mitochondria of Lake char (*Salvelinus namaycush*) with increasing temperature (Ballantyne *et al.,* 1989). Although teleost fishes do not rely as heavily on ketones as an extrahepatic fuel source (Zammit and Newsholme, 1979), a decrease in plasma ketones would correlate with an increase in ketone oxidation if the rate of hepatic ketogenesis does not increase in warmer temperatures in these groups of fishes. Elasmobranchs may utilize the same hepatic mechanisms and may not be able to maintain the production of 3-HB under these heatwave conditions.

There was an inverse trend between circulating glucose and 3-HB, where plasma glucose increased following air exposure, while 3-HB decreased. Similar inverse trends between glucose and β-HB have been observed in *S. acanthias suckleyi* during air exposure and confinement (Schoen *et al.*, 2021), and *S. canicula* seasonally (Gutiérrez *et al.,* 1987), suggesting that one metabolite may be preferential over the other during different stress events. However, air exposure in the Atlantic stingray (*Hypanus sabinus*) caused a rapid decrease in plasma glucose and no change in plasma β-HB (Lambert *et al.,* 2018), while electrical stimulation of perfused white muscle in *S. acanthias suckleyi* produced an influx of both metabolites (although the change in net flux of β-HB was significantly greater than glucose) (Richards *et al.,* 2003), postulating that the relationship between metabolites may be species and tissue specific. The deviation in both metabolites at 30 min in the present study indicates that air exposure further activated the secondary stress axis and presumably promoted the mobilization of glucose and consumption of 3-HB for at least 48 h, at which point both metabolites returned to baseline in both temperature treatments. The air exposure event in this study can be compared to a component of a capture event, either from targeted fishing or bycatch, which can cause species-specific physiological disturbances from forced exercise, physical trauma and periodic air exposure (Manire *et al.,* 2001; Skomal and Mandelman, 2012; Wilson *et al.,* 2014; Ellis *et al.,* 2017). This includes but is not limited to activation of the stress response, exhaustion and increased lactate/acidosis, changes in energy mobilization, changes in ion concentrations and cellular damage (Manire *et al.,* 2001; Wilson *et al.,* 2014), all of which must be resolved quickly to resume normal function when the fish is released. For instance, capture stress has been shown to increase glucose circulation in *C. melanopterus* (Bouyoucos *et al.,* 2018b), *N. brevirostris* (Bouyoucos *et al.,* 2017) and *R. terraenovae* (Hoffmayer and Parsons, 2001), and associated air exposure may exacerbate these effects further (Frick *et al.,* 2010). These effects can develop into long-term physiological and behavioural problems (Wilson *et al.,* 2014) under increased thermal stress, such as diverting energy away from growth and reproduction, thus perpetuating the effects of incidental catch.

The presumptive glucocorticoid in elasmobranchs is 1α-OHB (Hazon and Henderson, 1985; Anderson, 2012), and thus we predicted there would be an increase in 1α-OHB (and potentially corticosterone) following simulated stressors that correlated with the observed changes in metabolites. There was an increase in 1α-OHB between baseline levels and time-0 in both the 27°C- and 29°C -exposed sharks; although, this trend was not significant. As previously mentioned, *S. acanthias suckleyi* exhibited a similar increase in 1α-OHB following confinement stress (Schoen *et al.,* 2021). That may potentially have been the case in the present study as well, given that both the 27°C- and 29°C -exposed sharks experienced an increase in the hormone. There were significant differences between sharks sampled after 30 min and sharks sampled after 24 h and between 27°C- and 29°C-exposed sharks. Specifically, circulating 1α-OHB in 27°C-exposed sharks remained elevated when compared to levels measured in the 29°C-exposed sharks. Assuming the 27°C-exposed sharks represented control conditions, these data suggests that a rise in temperature caused a deviation in the expected corticosteroid and metabolite response in the 29°C-exposed sharks, which is further corroborated by the increase in corticosterone at 1 h in only the 27°C-exposed sharks. However, it is worth mentioning that plasma corticosterone concentrations in *C. melanopterus* are substantially lower than concentrations that have been documented in other species (Kime, 1977; Rasmussen and Gruber, 1993; Manire *et al.,* 2007; Brinn *et al.,* 2012, Schoen *et al.,* 2021). Bouyoucos *et al.* (2020) noted that the same population of *C. melanopterus* neonates experienced minor increases in metabolic rate if they had been acclimated to 31°C when compared to 28°C. However, these sharks were acclimated to elevated temperatures over a much longer time period (i.e. 4 weeks) (Bouyoucos *et al.,* 2020); whereas, the sharks in the present study were only briefly exposed (i.e. four days) to elevated temperatures, potentially indicating reduced metabolic hormone performance under a more acute thermal stress. In assessing the role of measured corticosteroids on energy mobilization under these circumstances, we did not determine significant correlations between 1α-OHB and corticosterone with either metabolite. Previously, a positive correlation between 1α-OHB and glucose in *S. canicula* was reported (Ruiz-Jarabo *et al.,* 2019); however, cortisol rather than 1α-OHB was used as a standard for steroid measurement, which may not provide the most relevant representation of circulating levels of the dominant elasmobranch corticosteroid, 1α-OHB. Furthermore, in the Pacific spiny dogfish (*S. acanthias suckleyi*), recent measurements of circulating levels of 1α-OHB (i.e. using a homologous standard) showed no relationship between plasma 1α-OHB and glucose (Schoen *et al.,* 2021). Following the results on *S. acanthias suckleyi*, a possible glucocorticoid action of corticosterone has been suggested, but in the present study, corticosterone concentrations in *C. melanopterus* were too low to indicate a correlation.

### Wild-caught sharks (neonates versus adults)

This is the first study that concurrently measured 1α-OHB, corticosterone, glucose and 3-HB in both neonates and adults of the same elasmobranch species, giving insight into hypothalamic–pituitary–interrenal (HPI) axis development. Across both metabolites, there was little change between timepoints within age class, indicating that 5 min of handling may not have been long enough to mount a measurable difference in the secondary stress markers. However, there was a significant increase in 1α-OHB in neonates between time-0 and 5 min. Additionally, there were significant increases in glucose and 1α-OHB concentrations between neonates and adults at both timepoints, where adults had lower glucose and higher 1α-OHB concentrations than neonates, which may be an effect of age/sexual maturity and/or habitat choices. In teleost fishes, the complete development of the HPI axis can be delayed for several days to weeks post-hatch (Barry *et al.,* 1995; Wendelaar Bonga, 1997; Jentoft *et al.,* 2002; Alsop and Vijayan, 2008; Earhart *et al.,* 2020). In the present study, while we could not assign a specific age to either the neonates or adults, all neonates captured had a visible umbilical scar, indicating they had been born approximately within 4 weeks (Chin *et al.,* 2015). Ontogeny of the HPI axis has not been specifically examined in elasmobranchs, but it may be that elasmobranchs follow a similar delay that has been described in some teleosts, and therefore would be unable to mount a vigorous corticosteroid response. Furthermore, neonates inhabit nearshore environments that provide protection from predators and potentially increased access to prey (Heupel *et al.,* 2007; Knip *et al.,* 2010; Schlaff *et al.,* 2014; Oh *et al.,* 2017). However, the neonate environment may be inherently more stressful due to highly variable abiotic conditions, particularly temperature (Heupel *et al.,* 2007; Knip *et al.,* 2010), which may increase energetic demands (Schlaff *et al.,* 2014). Neonates may compensate for the dynamic abiotic conditions in their habitat with a muted endocrine stress response such that energy can be directed towards growth. Additionally, an increase in 1α-OHB has been previously related to fasting in the Japanese banded houndshark (*Triakis scyllium*; Iki *et al.,* 2020), which implies that 1α-OHB concentrations could also be related to feeding status of neonate sharks. In fact, drastic shifts in energy pathways were recorded in *C. melanopterus* during the first year of life based on stable isotope tissue analysis in relation to total body length (Matich *et al.,* 2015). Matich *et al.* (2015) attributed this shift to changing trophic levels when sharks switch from acquiring energy from primarily hepatic stores to foraging, which would account for higher glucose concentrations in neonates if the principal energy sources were derived from hepatic glycogenolysis and gluconeogenesis.

### Laboratory versus wild neonates

Laboratory-based neonates (27°C) demonstrated higher 3-HB and 1α-OHB than field neonates at time-0, which may be a reflection of 6 days of confinement. Previous studies found that spiny dogfish, *S. acanthias* (Mandelman and Farrington, 2007), *S. acanthias suckleyi* (Schoen *et al.,* 2021) and *S. canicula* (Torres *et al.,* 1986), exhibited signs of the secondary stress response and an increase in circulating glucose following confinement stress. Additionally, Schoen *et al.* (2021) demonstrated that there was also an increase in circulating 1α-OHB and β-HB following confinement in *S. acanthias suckleyi*. Confinement stress may be acting as a simultaneous and persistent stressor in the laboratory-maintained animals in this study. Although, there was no difference in circulating glucose between wild and laboratory-based neonates, indicating that the stress axis may not have been stimulated sufficiently to elicit glucose mobilization or that these animals may not be predominately relying on glucose to fuel a sustained stress response. Furthermore, variations in β-HB and 1α-OHB levels have been previously associated with feeding status in elasmobranchs. Specifically, fasting can induce an increase in plasma β-HB levels in *S. acanthias suckleyi* (Wood *et al.,* 2010 and plasma 1α-OHB levels in *T. scyllium* (Iki *et al.,* 2020). Given that we do not know the feeding status of the wild neonates, there may be discrepancies in baseline measurements between laboratory-maintained and wild sharks that were influenced by degree of satiation.

## Conclusions

Nearshore habitats, particularly shallow tropical environments, inherently experience dynamic abiotic conditions, but are also expected to be drastically impacted by increasing ocean temperatures with climate change (Collins *et al.,* 2013). These habitats are essential for neonate sharks, acting in some cases as nursery areas, providing refuge from predators, and potentially access to prey. In the present study, a 2°C temperature increase affected the secondary stress response of *C. melanopterus* neonates. Specifically, neonates experienced a predictable rise in glucose, a decline in 3-HB, and suppression of the dominant corticosteroid, 1α-OHB. In addition, we revealed differences in circulating glucose and 1α-OHB levels between neonate and adult sharks, which may signal shifts in ontogenetic endocrine and/or energy partitioning during the acute stage of the stress response.

It is critical to understand the underlying mechanisms of the stress response in near-future temperature conditions. The narrow thermal tolerance range that many tropical reef fishes have evolved (Rummer *et al.,* 2014; Rummer and Munday, 2017) places them at significant risk of thermal stress both now and into the future. Our results indicate that thermal stress will likely affect corticosteroid-related functions and energy balance in this species, which may result in increased susceptibility to additional stressors such as environmental change, fishing pressure, and predation. To our knowledge, this is the first study to examine temperature effects on hormonal regulation of energy balance in elasmobranchs. As such, this data contributes to the overall understanding and assessment of 1α-OHB as a stress biomarker in sharks. However, changes in glucocorticoid and secondary (energy) metabolites in response to the simulated stressors was variable, and more complex than expected. We conclude that 1α-OHB may not be a reliable tool in measuring the stress response in neonate *C. melanopterus* under these conditions. Additional work is needed to characterize the acute and chronic phases of the stress response in order to determine if 1α-OHB has a role in energy mobilization under different timescales. In the meantime, measuring secondary stress markers, such as glucose, remains the most acceptable method in evaluating stress in sharks until further research confirms the energy-regulating role of corticosteroids in this group of fishes.

## Funding

This work was supported by The Company of Biologists and Journal of Experimental Biology Travel Award awarded to A.N.S., an NSERC Discovery grant awarded to W.G.A. (05348-15) and an ARC Discovery Early Career Researcher Award (PDE150101266) awarded to J.L.R.

## Conflicts of Interest

The authors declare no conflicts of interest.
